# Individualized dynamic PEEP (dynPEEP) vs. positive pressure ventilation in delivery room management: A retrospective cohort study

**DOI:** 10.3389/fped.2022.1007632

**Published:** 2023-01-11

**Authors:** Sijie Song, Yefang Zhu, Jie Li, Qi Wang, Hua Gong, Xiaoyun Zhong, Yan Wu

**Affiliations:** ^1^ Department of Pediatrics, Women and Children's Hospital of Chongqing Medical University, Chongqing, China; ^2^ Department of Pediatrics, Chongqing Health Center for Women and Children, Chongqing, China

**Keywords:** dynamic PEEP, positive pressure ventilation, bronchopulmonary dysplasia, intubation, delivery room management

## Abstract

**Objective:**

Although nasal continuous positive airway pressure (nCPAP) is recommended in delivery room (DR) management for preterm infants, the effect of delivering nCPAP at 6–8 cmH_2_O is not satisfactory. Therefore, we conducted this retrospective cohort study to compare the effects of individualized dynamic positive end-expiratory pressure (dynPEEP) vs. positive pressure ventilation (PPV) in the DR on clinical outcomes.

**Methods:**

Preterm infants with a gestational age (GA) less than 30 weeks who received PPV (peak inspiratory pressure, PIP/PEEP 15–25/6–8 cmH_2_O) from August 2018 to July 2020 were included as Cohort 1 (PPV group, *n* = 55), and those who received dynPEEP (nCPAP 8–15 cmH_2_O) from June 2020 to April 2022 were included as Cohort 2 (dynPEEP group, *n* = 62). Primary outcomes included the DR intubation rate and the bronchopulmonary dysplasia (BPD) rate. The secondary outcomes included DR stabilization, transfer, admission, respiratory function, and other outcomes.

**Results:**

The percentage of singleton infants was higher in the PPV group (63.6%) than in the dynPEEP group (22.6%, *p* = 0.000). The DR intubation and chest compression rates were higher in the PPV group (80.0% and 18.2%, respectively) than in the dynPEEP group (45.2%, *p* = 0.000; 3.0%, *p* = 0.008, respectively). The percentage of patients with 5-min Apgar scores < 5 was higher in the PPV group (9.1%) than in the dynPEEP group (0%, *p* = 0.016). The partial pressure of carbon dioxide was lower in the PPV group (49.77 ± 11.28) than in the dynPEEP group (56.44 ± 13.17, *p *= 0.004), and lactate levels were higher in the PPV group (3.60 (2.10, 5.90)) than in the dynPEEP group (2.25 (1.38, 3.33), *p *= 0.002). No significant differences in the BPD rate or other secondary outcomes were noted.

**Conclusions:**

In this retrospective cohort study, the dynPEEP strategy reduced the need for DR intubation compared with PPV. The dynPEEP strategy is feasible and potentially represents an alternative respiratory strategy to PPV. Nevertheless, a randomized control trial is needed to evaluate the dynPEEP strategy.

## 1. Background

Some preterm infants require respiratory support at birth to adequately aerate their lungs (1–5). Lung aeration plays an important role in the transition from intrauterine to extrauterine life. However, studies have demonstrated a direct relationship between exposure to intubation (and/or mechanical ventilation, MV) and an increased risk of developing bronchopulmonary dysplasia (BPD) (6, 7). Therefore, noninvasive respiratory support in the form of nasal continuous positive airway pressure (nCPAP) of 6–8 cmH_2_O is recommended in the international guidelines for delivery room (DR) management (8, 9). Theoretically, nCPAP could contribute to early physiological transition by facilitating alveolar recruitment and establishing functional residual capacity in preterm infants. However, although we performed nCPAP for all very preterm infants (VPI: gestational age less than 32 weeks) immediately after birth, 17% of VPIs and 36% of extremely preterm infants (EPI: gestational age less than 28 weeks) required intubation in the DR (10).

Using higher pressures up to 20–25 cm H_2_O for a period of approximately 10–15 s at the initiation of respiration (sustained lung inflation, SLI) has been reported as a method to avoid intubation (11). However, the Sustained Inflation of Infants Lung (SAIL) trial was suspended early because it detected higher rates of early death, possibly attributable to receiving SLI (20–25 cmH_2_O, 10–15 s) (12). The European Guidelines on Respiratory Distress Syndrome (RDS) management recommend that SLI should only be used in clinical trials until further analysis of available data (8). Preclinical studies among premature lambs have shown that rapidly aerating the preterm lung at birth produces distinct regional injury patterns that affect subsequent tidal ventilation (13). The ongoing development and heterogeneity of the preterm lung are not conducive to rapid forced aeration (e.g., SLI) (14). On the other hand, SLI before tidal ventilation is not physiological in the preterm lung, and SLI at birth blunted the effect of surfactant on lung compliance (15).

However, to date, the ideal level of nCPAP in the DR remains unknown. Clinical studies regarding the appropriate positive end-expiratory pressure (PEEP) levels, which could be provided by nCPAP, are lacking. Te Pas et al. reported that immature rabbits required higher starting pressures and longer sustained inflation durations to achieve a set inflation volume (16). Higher PEEP levels improve lung aeration and pulmonary blood flow and reduce the need for positive pressure ventilation (PPV) (17). A retrospective study on EPIs showed that nCPAP with higher pressures (12–35 cmH_2_O) at birth may require less oxygen and decrease intubation rates compared to pressures of 5–8 cmH_2_O, whereas the pneumothorax rates (19 vs. 4%, *p* = 0.125) and the occurrence of spontaneous intestinal perforations (15 vs. 0%, *p* = 0.125) were increased (18). This study indicated that initial respiratory support for EPIs with high nCPAP levels might decrease intubation rates. However, the adverse event rates increased, and the optimal nCPAP pressure was not specified.

A longitudinal study indicated that after the introduction of a revised protocol to assist EPIs with a GA of 22–26 weeks, the rates of infants intubated in the DR and BPD were significantly decreased (19). In this study, the revised PEEP protocol used in the DR was between 8 and 14 cm H_2_O, and no harmful effects were noted. Instead, the overall mortality in the revised study group was lower than that in the control group. This study may imply that using a PEEP level of 8–14 cm H_2_O would be beneficial for EPIs. However, the protocol includes several interventions [e.g., prenatal management and delayed cord clamping (DCC)]; thus, the beneficial outcomes are not only associated with the revised PEEP protocol.

Above all, we hypothesize that different preterm infants may have different lung development conditions; thus, the PEEP required at birth to adequately aerate their lungs is different. In addition, the intubation rate is still high in the DR, which may also indicate that the effect of delivering nCPAP of 6–8 cmH_2_O is not satisfactory. Therefore, an individualized dynamic PEEP (dynPEEP) is proposed. DynPEEP using an optimal PEEP strategy might be more lung protective than SLI because it adjusts the pressure required according to the vital signs of preterm infants. In preterm lambs, dynPEEP at birth with tidal ventilation and PEEP results in more uniform aeration and ventilation and less lung injury than SLI (13) and improves the surfactant response (15). We inferred that the improved surfactant response using the dynPEEP strategy, which increased functional residual capacity and improved lung compliance/homogeneity compared with SLI, would be more likely to reduce the incidence of PPV and intubation. A recent study conducted in France showed that dynPEEP combined with the SLI strategy was beneficial. However, it is difficult to explain whether the benefit is attributable to dynPEEP or SLI (20).

The dynPEEP strategy was implemented in our hospital in June 2020. We conducted this retrospective cohort study to compare the dynPEEP strategy with PPV in the DR on the clinical outcomes of preterm infants with a gestational age (GA) less than 30 weeks who did not respond to an initial CPAP at 6–8 cmH_2_O.

## 2. Methods

### 2.1. Inclusion and exclusion criteria

All inborn infants < 30 weeks of gestation admitted to the neonatal intensive care unit (NICU) of the Women and Children's Hospital of Chongqing Medical University, which is a tertiary hospital in southwestern China, were initially included in this study. The study period was between August 2018 and April 2022.

The inclusion criteria were as follows: 1. Infants delivered at <30 weeks of gestation who did not respond to an initial CPAP at 6–8 cmH_2_O; and 2. noninvasive respiratory support was provided immediately after birth in the DR.

The exclusion criteria were as follows: 1. Refusal of consent for the data to be analyzed; 2. there was no need for any respiratory support or only support with a PEEP of 6–8 cmH_2_O in the DR, but dynPEEP or PPV was not provided; 3. known major congenital anomalies or inherited metabolic diseases that might have an adverse effect on breathing or ventilation; 4. maternal factors, such as general anesthesia, placental abruption, placenta previa, and monochorionic twins; and 5. outborn infants.

### 2.2. Participants

Two cohorts of preterm infants born at < 30 weeks of gestation and observed for 4 years were compared retrospectively before (Cohort 1: PPV group) and after the renovation of respiratory support management in the DR (Cohort 2: dynPEEP group). In Cohort 1, preterm infants who received PPV (peak inspiratory pressure, PIP/PEEP 15–25/6–8 cmH_2_O) from August 2018 to July 2020 were included (the PPV group, *n* = 55). In Cohort 2, preterm infants who received dynPEEP (nCPAP 8–15 cmH_2_O) from June 2020 to April 2022 were included (the dynPEEP group, *n* = 62).

### 2.3. Stabilization and respiratory support in the DR

All team members were trained to deliver noninvasive respiratory support. At delivery, DCC was performed first. The obstetricians and/or midwife were the DCC providers, and they simultaneously gently rubbed the backs of the infants. The delay time of DCC was based on the recommendation made by the European Consensus Guidelines and the American Heart Association to wait for at least 30–60 s (8,9). Then, eligible infants (23^0/7^ to 29^6/7^ weeks GA) were placed onto a radiant warmer (Giraffe Omnibed) wrapped in plastic wrap. Then, nCPAP was given *via* a face mask or nasal prong using a T-piece device (Neopuff Infant Resuscitator, Fisher & Paykel Healthcare, Auckland, New Zealand) with pressure at 6–8 cmH_2_O and a fraction of inspired oxygen (FiO_2_) of 21%–30% (30% for babies < 28 weeks gestation and 21%–30% for those 28–30 weeks gestation) (8). A pulse oximeter probe (L-NOPNeo; Masimo Corp, Irvine, CA) was placed on the right hand, and 3 ECG chest leads were applied on the chest. The oximeter was set to acquire data with maximum sensitivity and a 5 s average interval.

In the PPV group, if the infant was apneic (no spontaneous breathing with stimulation by rubbing the soles of the feet or the back of the chest for more than 10 s) and/or bradycardic (heart rate less than 100 bpm), PPV (peak inspiratory pressure, PIP/PEEP 25/6%–8% cmH_2_O, 40–60/min) was administered. The FiO_2_ was initially set at 30% and could be adjusted to 100% based on the 25th percentile of the Dawson criteria (21). Respiratory support was provided using the Neopuff TM T-piece resuscitator (Neopuff Infant Resuscitator, Fisher & Paykel Healthcare Ltd., Auckland, New Zealand) *via* face mask or prongs.

**Figure 1:**
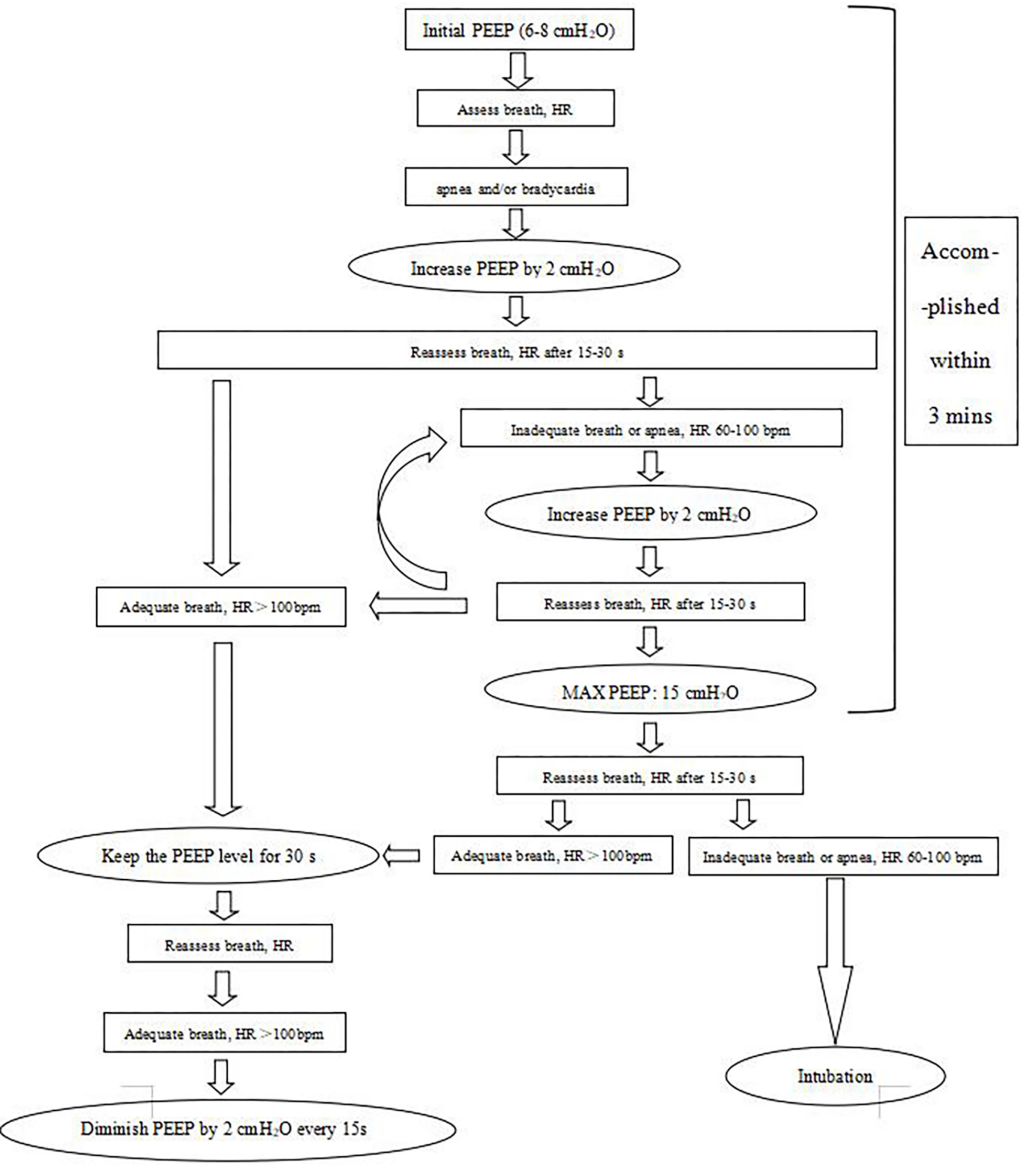
The dynPEEP algorithms in DR management.

In the dynPEEP group, if the infant was apneic and/or bradycardic (same definition as mentioned above), PEEP was increased by 2 cmH_2_O/15–30 s to a maximum of 15 cmH_2_O without any PIP inflation. The FiO_2_ was initiated at 30% and could be increased to 100% based on the 25th percentile of the Dawson criteria. The PEEP was increased prior to FiO_2_ initiation. PEEP was allowed to be decreased if a baby improved. Figure 1 shows the dynPEEP algorithms in DR management. To ensure that this new method would be adopted by our staff, we trained the staff first at the beginning of the implementation. It took approximately 2 months (from the beginning of May 2020 to the end of June) for all the staff to adhere to this new method. At the beginning, all the stabilization processes of preterm infants with GAs less than 30 weeks were videotaped (the first 3 weeks in May), and the resuscitation records and videos were used to check whether the dynPEEP algorithms were carried out. We were then debriefed on the stabilization process and gave feedback to the staff who were in charge, and we trained the staff repeatedly. At the end of May 2020, the video playback and debrief process were performed approximately once or twice a week with the gradual acceptance of this new intervention. At the end of June 2020, approximately 90% of the providers stabilized the infants according to the dynPEEP algorithms in the DR.

If PPV or dynPEEP was ineffective, then the infant was intubated. The DR intubation criteria were as follows: (1) heart rate less than 60 bpm after 30 s of effective respiratory support (dynPEEP or PPV; in this case, dynPEEP was interrupted at 10–12 cmH_2_O), (2) heart rate remaining at less than 100 bpm 3 min after birth, and (3) still apneic 3 min after birth. Intubation was performed at the discretion of the neonatologist in charge.

The less invasive surfactant administration (LISA) method was used for pulmonary surfactant administration in the DR for all infants in case intubation was not needed. The LISA method was introduced in our unit in 2017 as a priority method for all preterm infants less than 1500 grams or with a GA less than 32 weeks. All the doctors who used this method were highly trained and experienced. If the infant was already intubated, then the intubation-surfactant-extubation (INSURE) procedure or intubation-surfactant (INSUR) without the extubation method was used, which were introduced in our unit in 2010. Surfactant was administered through the endotracheal tube; if the FiO_2_ dropped to 30% in a short time without dyspnea, then extubation and noninvasive respiratory support were continued. If not, MV was implemented. Pulmonary surfactant was used in the DR or in the NICU.

After stabilization in the DR, the infants were transported to the NICU in incubators. We used a Hamilton ventilator (Hamilton Medical AG, Switzerland) during the transfer for both noninvasive and mechanical ventilation. If the infant was on noninvasive respiratory support (nCPAP or nasal intermittent positive pressure ventilation, NIPPV), then a binasal prong was used as the interface. The methods and settings of respiratory support during the transfer were recorded in the resuscitation records.

In the NICU, the criteria for intubation were as follows: (1) for infants <32 weeks gestation with severe apnea (defined as recurrent apnea with >3 episodes/h associated with a heart rate < 100/min, a single episode of apnea that required PPV, or saturation of pulse oxygen (SpO_2_) < 85% and FiO_2_ > 0.6); (2) for infants with RDS or dyspnea that rapidly progressed and/or persisted after noninvasive ventilation and/or pulmonary surfactant treatment, and FiO_2_ ≥ 40%, PaO_2_ < 50–60 mmHg or SpO_2_ < 90% (except for cyanotic heart disease), or partial pressure of carbon dioxide (PCO_2_) > 60–65 mmHg, pH <7.25; and (3) if there was some instability in the hemodynamics of the infants. The extubation criteria were as follows: extubation was recommended within 24 h of meeting all the following criteria: PCO_2_ of 55 mmHg or less, pH of 7.25 or greater, FiO_2_ of 0.40 or less with oxygen saturation as measured by pulse oximetry (SpO_2_) of 90% or greater, and mean airway pressure (MAP) of 8 cmH_2_O or less with hemodynamic stability. High-frequency oscillation (HFO) ventilation was recommended if one of the following criteria was met: (1) PCO_2_ > 60–65 mmHg and pH <7.25 even when the tidal volume reached 6 ml/kg and the minute volume reached 0.3 L/min under MV, (2) excess secretion in the airway, and (3) air leakage, including pneumothorax and mediastinal emphysema. Permissive hypercapnia (pH >7.25, pCO_2_ 50–65 mmHg) was allowed in our unit. The criteria to start systemic corticosteroids were as follows: (1) for infants with severe BPD according to the National Institute of Child Health and Human Development (NICHD) workshop definition (22), (2) for infants who required MV for more than 7–10 days since birth, and for those who had FiO_2_ > 0.4. Systematic sedation-analgesia (fentanyl and/or midazolam) was used only for mechanically ventilated infants who were irritated. The doses of caffeine citrate were as follows: a loading dose of 20 mg/kg and a maintenance dose of 5 mg/kg q24 h or q12 h.

### 2.4. Demographic characteristics and clinical outcomes

#### 2.4.1. Demographic characteristics

All data on neonatal and maternal demographics were collected *via* resuscitation and electronic medical records.

#### 2.4.2. Primary outcomes

The primary outcomes were the DR intubation rate and bronchopulmonary dysplasia (BPD) rate.

The definition of BPD was as follows: A premature infant (<32 weeks gestational age) with BPD had persistent parenchymal lung disease, radiographic confirmation of parenchymal lung disease, and required respiratory support or oxygen mode for ≥ 3 consecutive days to maintain arterial oxygen saturation in the 90%–95% range at 36 weeks PMA (22).

#### 2.4.3. Secondary outcomes

1) *DR outcomes*

The DR chest compression rate; Apgar scores at 1, 5, and 10 min; Apgar score < 1 at 1 min and < 5 at 1, 5, and 10 min; and DR maximum fraction of inspired oxygen (FiO_2_) were the DR outcomes.

2) *Transfer outcomes: Respiratory support during the transfer to the NICU*

The methods and settings of respiratory support during the transfer were collected from the resuscitation records.

3) *Admission outcomes*

Admission arterial blood gas, including pH values, partial pressure of oxygen, partial pressure of carbon dioxide, base excess, lactate, maximum FiO_2_, and *P*/F (formula: PaO_2_ ÷ FiO_2_).

4) *Respiratory outcomes*

Mortality within 48 h after birth, surfactant therapy, surfactant administration ≥ 2 times, pneumothorax rate within 72 h of age, MV within 72 h of age, MV during hospitalization, time of start on MV (hours), duration of MV (hours), duration of noninvasive respiratory support (hours), duration of oxygen therapy (days), systematic dexamethasone and treated PDA (ibuprofen), mortality and composite BPD and/or mortality rate were the respiratory outcomes.

5) *Other outcomes*

Early-onset sepsis (EOS) (23), late-onset sepsis (LOS) (23), necrotizing enterocolitis (NEC) (≥ phase 2) (24), intraventricular hemorrhage (IVH) (≥ grade 3) (25), and retinopathy of prematurity (ROP) (> phase 2) were the other outcomes (26).

All data on outcomes were collected *via* resuscitation and electronic medical records. Outcomes with missing data due to transfer or death prior to assessment were excluded. Both the number of infants with the outcome and the number assessed are shown.

### 2.5. Data analysis

Data were analyzed using IBM SPSS Statistics version 23.0 (IBM Software, Chicago, Illinois, United States, 2016). The normality test and the homogeneity of variance test were performed for continuous data. If the data were normally distributed and homogenous in variance, the data were expressed as the means ± standard deviations (*X* ± SDs), and Student's t test was used for comparisons between the two groups. If the data were not normally distributed or the variance was not uniform, the rank-sum test (Mann‒Whitney *U* test) was used, and the data were presented as the medians (interquartile ranges, IQRs). The categorical data were expressed as percentages (%), and the rates were compared between the two groups by the chi-square test or Fisher's exact probability method and are presented as counts (*n*) and percentages (%). To control for the confounders for DR intubation, the demographic characteristics and variables with a *P* value < 0.05 in the univariate analysis were applied to the multivariate analysis based on logistic regression. The power of the association was represented by *χ*^2^ in the logistic regression model. Furthermore, a subgroup analysis was performed for infants with a GA less than 28 weeks. *P* values < 0.05 were considered statistically significant, and reported *P* values were two sided. No adjustment of *P* values was performed to account for multiple comparisons because subgroup analyses were considered exploratory.

### 2.6. Ethics

This study was approved by the Ethics Committee of Chongqing Health Center for Women and Children (No. 2021–022).

## 3. Results

Of the 377 preterm infants who were potentially eligible to participate, 117 were included in this study, and 260 did not meet our inclusion criteria. In total, 55 infants were supported with PPV, and 62 infants were supported with dynPEEP.

**Figure 2:**
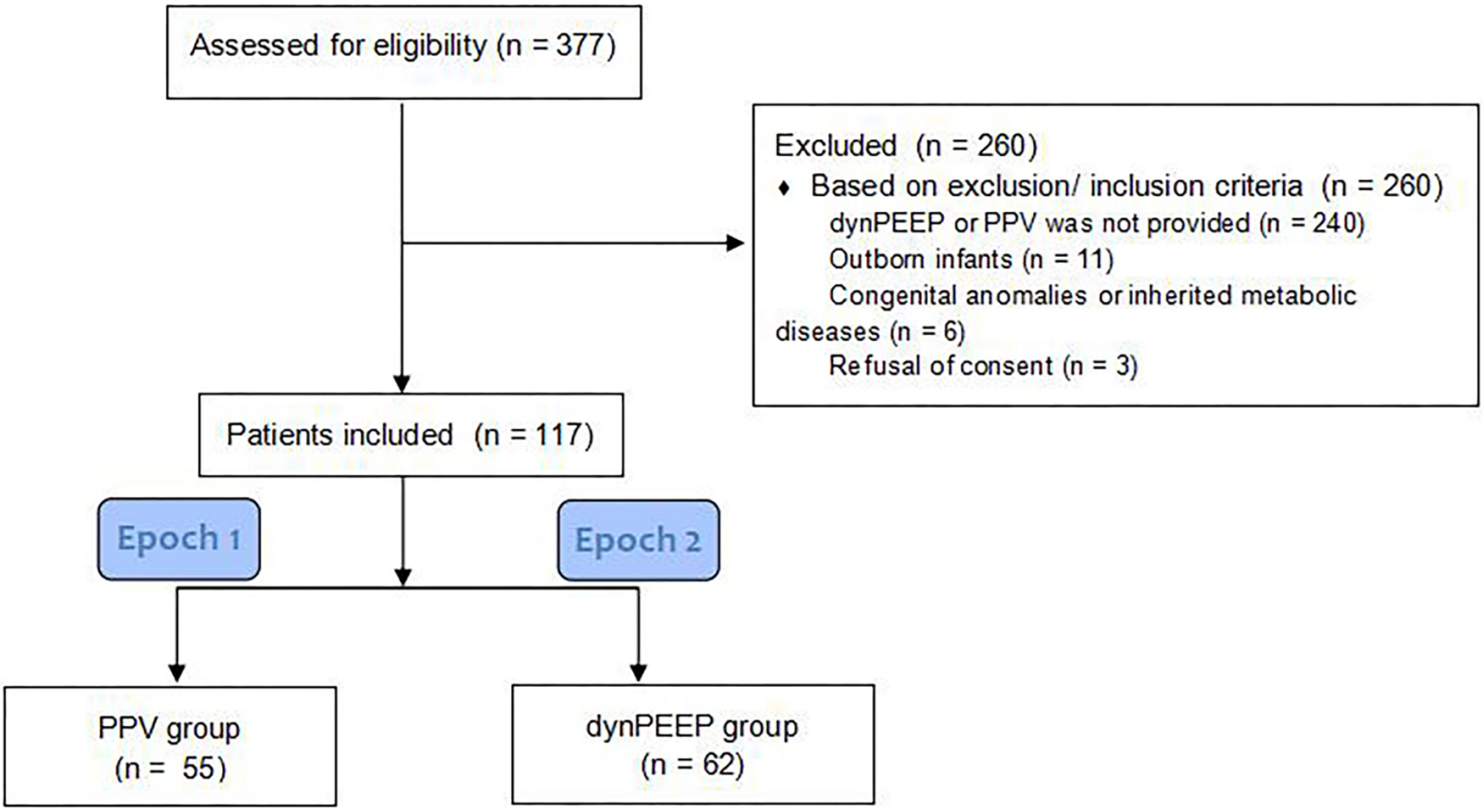
Flowchart of patient inclusion.

The flowchart shows the inclusion and exclusion criteria for the patients (Figure 2).

### 3.1. Maternal and neonatal demographics

The maternal and neonatal demographics are shown in Table 1. The percentage of singleton infants in the PPV group (63.6%) was significantly higher than that in the dynPEEP group (22.6%, *p* = 0.000). There were no significant differences in the other demographics between the two groups.

**Table 1:** Baseline maternal and neonatal demographic and clinical characteristics.

	PPV (*n* = 55)	dynPEEP (*n* = 62)
Neonatal Demographics		
Gestational age, (*x *± SD)	27.7 ± 1.6	27.2 ± 1.5
Birthweight, (*x *± SD)	1050 ± 237	997 ± 239
Male sex, *n* (%)	34 (61.8%)	30 (48.4%)
Singleton birth, *n* (%)	35 (63.6%)	14 (22.6%)*
Cesarean delivery, *n* (%)	39 (70.9%)	35 (56.5%)
SGA, *n* (%)	2 (3.6%)	1 (1.6%)
DCC, *n* (%)	35 (63.6%)	47 (75.8%)
Maternal Demographics		
Pregnancy-induced hypertension, *n* (%)	12 (21.8%)	9 (14.5%)
GDM, *n* (%)	14 (25.5%)	16 (25.8%)
ICP, *n* (%)	0	0
PROM, *n* (%)	16 (29.1%)	19 (30.6%)
Chorioamnionitis, (*n*) (%)	4 (7.3%)	7 (11.3%)
Antenatal steroids (full course), *n* (%)	29 (52.7%)	41 (66.1%)
Antenatal magnesium sulfate, *n* (%)	37 (67.3%)	45 (72.6%)
Antenatal antibiotics, *n* (%)	18 (32.7%)	27 (43.5%)

*: vs. PPV group, *P* < 0.01.

IQR, interquartile range; SGA, Small for gestational age; DCC, delayed cord clamping; GDM, gestational diabetes mellitus; ICP, intrahepatic cholestasis of pregnancy; PROM, premature rupture of membranes; PPV, positive pressure ventilation; dynPEEP, dynamic positive end expiratory pressure.

The DR maximum PEEP was significantly higher in the dynPEEP group (12, (10, 15)) than in the PPV group (6, (6, 8)) (*p* = 0.000). In the dynPEEP group, the number and percentage of cases with different PEEP levels were 21 cases (33.87%) at 9–10 cmH_2_O, 25 cases (40.32%) at 11–14 cmH_2_O, and 16 cases (25.81%) at 15 cmH_2_O.

### 3.2. Primary outcomes

The DR intubation rate was higher in the PPV group (80.0%) than in the dynPEEP group (45.2%, *p* = 0.000) (Table 2). No significant difference in the BPD rate was noted between the two groups.

**Table 2:** The primary outcomes of preterm infants with gestational Age less than 30 weeks who received PPV or DynPEEP in the DR.

	PPV (*n* = 55)	dynPEEP (*n* = 62)	*P* value
**Primary Outcomes**			
DR intubation rate, *n* (%)	44 (80.0%)	28 (45.2%)	0.000
BPD, *n* (%)	14/43 (32.6%)	13/45 (28.9%)	0.709
***Subgroup analysis (GA less than 28 weeks)***	(*n* = 26)	(*n* = 45)	
DR intubation rate, *n* (%)	22 (84.6%)	24 (53.3%)	0.008
BPD, *n* (%)	10/18 (55.6%)	13/29 (44.8%)	0.474

DR, delivery room; BPD, bronchopulmonary dysplasia; PPV, positive pressure ventilation; dynPEEP, dynamic positive end expiratory pressure.

Note: For the calculation of BPD, the denominator indicates the number of preterm infants who survived at 36 GA in our NICU. Those who died or were transferred to another hospital prior to assessment were excluded. Both the number of infants with the outcome and the number assessed are shown.

### 3.3. Secondary outcomes

#### 3.3.1. DR, transfer, and admission outcomes

Table 3 displays the DR, transfer, and admission outcomes.

**Table 3:** The secondary outcomes (DR, transfer, and admission) of preterm infants with gestational Age less than 30 weeks who received PPV or DynPEEP in the DR.

	PPV (n = 55)	dynPEEP (n = 62)	*P* value
**Secondary Outcomes**			
**DR outcomes**			
DR chest compression rate, *n* (%)	10 (18.2%)	2 (3.2%)	0.008
Apgar scores			
< 3 at 1 min, *n* (%)	7 (12.7%)	3 (4.8%)	0.129
< 5 at 1 min, *n* (%)	19 (34.5%)	15 (24.2%)	0.218
< 5 at 5 min, *n* (%)	5 (9.1%)	0	0.016
< 5 at 10 min, *n* (%)	2 (3.6%)	0	0.132
Maximum DR FiO_2_, median (IQR)	70 (45, 100)	60 (45, 100)	0.372
**Transfer outcomes**			
Respiratory mode			
nCPAP, *n* (%)	16 (29.1%)	32 (51.6%)	0.035
NIPPV, *n* (%)	4 (7.3%)	5 (8.1%)	
MV, *n* (%)	35 (63.6%)	25 (40.3%)	
Settings			
nCPAP			
FiO_2_	30 (25, 40)	30 (26, 39)	0.797
PEEP	6 (6, 7.5)	8 (7, 8)	0.003
MV			
FIO_2_	35 (30, 45)	40 (30, 50)	0.554
PIP	20 (16, 20)	16 (15.5, 18)	0.016
PEEP	6 (6, 6)	6 (6, 8)	0.025
**Admission outcomes**			
pH values, (*x *± SD)	7.23 ± 0.08	7.20 ± 0.09	0.108
PaO_2_, (*x* ± SD)	89.2 ± 26.5	89.5 ± 30.3	0.956
PaCO_2_, (*x* ± SD)	49.8 ± 11.3	56.4 ± 13.2	0.004
Base excess, (*x* ± SD)	−6.6 ± 4.1	−6.4 ± 3.7	0.714
Lactate, median (IQR)	3.6 (2.1, 5.9)	2.3 (1.4, 3.3)	0.002
Maximum FiO_2_, median (IQR)	35 (30, 45)	35 (25, 45)	0.954
*P*/F, median (IQR)	245 (180, 349)	286 (200, 350)	0.346

IQR, interquartile range; DR, delivery room; FiO_2_, fraction of inspired oxygen; PaO_2_, partial arterial oxygen pressure; PaCO_2_, partial arterial carbon dioxide pressure; nCPAP, nasal continuous positive airway pressure; NIPPV, nasal intermittent positive pressure ventilation; MV, mechanical ventilation; PIP, peak inspiratory pressure; PEEP, positive end expiratory pressure; PPV, positive pressure ventilation; dynPEEP, dynamic positive end expiratory pressure.

Note: *P*/F (formula: PaO_2_ ÷ FiO_2_).

For the DR outcomes, the DR chest compression rate was higher in the PPV group (18.2%) than in the dynPEEP group (3.2%, *p* = 0.008), and the percentage of patients with 5 min Apgar scores < 5 at was higher in the PPV group (9.1%) than in the dynPEEP group (0%, *p* = 0.016).

With regard to the transfer outcomes, the percentage of infants with noninvasive respiratory support in the dynPEEP group was higher than that in the PPV group. The settings during the transfer also exhibited significant differences in PEEP levels (nasal or MV) and PIP levels (MV) between the two groups.

Regarding the admission outcomes, the partial pressure of carbon dioxide on admission was lower in the PPV group (49.8 ± 11.3) than in the dynPEEP group (56.4 ± 13.2, *p* = 0.004), and the lactate levels were higher in the PPV group (3.6 (2.1, 5.9)) than in the dynPEEP group (2.3 (1.4, 3.3), *p* = 0.002).

No significant differences in the other DR, transfer, or admission outcomes were noted.

#### 3.3.2. Respiratory and other outcomes

Table 4 displays respiratory and other outcomes. The percentage of the less invasive surfactant administration (LISA) method was lower in the PPV group (25.5%) than in the dynPEEP group (50%, *p* = 0.006). No differences in other secondary respiratory or other outcomes were noted between the two groups, including mortality within 48 h after birth, mortality rate, composite BPD and/or mortality rates.

**Table 4:** The secondary outcomes (respiratory and other) of preterm infants with gestational Age less than 30 weeks who received PPV or DynPEEP in the DR.

	PPV (*n* = 55)	dynPEEP (*n* = 62)	*P* value
**Secondary Outcomes**			
**Respiratory outcomes**			
Mortality within 48 h after birth, *n* (%)	2 (3.6%)	4 (6.5%)	0.491
Surfactant, *n* (%)	51 (92.7%)	58 (93.5%)	0.861
LISA method	14 (25.5%)	31 (50.0%)	0.006
INSURE method	14 (25.5%)	8 (12.9%)	0.083
Surfactant ≥ 2 times, *n* (%)	9 (16.4%)	15 (24.2%)	0.295
Pneumothorax within 72 h of age, *n* (%)	2 (3.6%)	0	0.130
MV within 72 h of age, *n* (%)	29 (52.7%)	25 (40.3%)	0.179
MV during hospitalization, *n* (%)	29 (52.7%)	32 (51.6%)	0.904
Time start on MV (h), median (IQR)	0.5 (0.5, 4.5)	0.75 (0.2, 32.3)	0.728
Duration of MV (h), median (IQR)	72 (28.5, 144.5)	64.5 (15.9, 137.5)	0.544
Duration of non-invasive respiratory support (h), median (IQR)	216 (120.0, 427.0)	239 (93.3, 490.3)	0.842
Duration of oxygen therapy (d), median (IQR)	38.7 (18.3, 51.9)	34.6 (11.5, 59.1)	0.816
Systemic dexamethasone, *n* (%)	4 (7.3%)	1 (1.6%)	0.186
Treated PDA (Ibuprofen), *n* (%)	14 (25.5%)	19 (30.6%)	0.533
Mortality, *n* (%)	9 (16.4%)	9 (14.5%)	0.782
Composite BPD and/or mortality, *n* (%)	21 (38.2%)	22 (35.5%)	0.763
**Other outcomes**			
Early-onset sepsis, *n* (%)	12 (21.8%)	13 (21%)	0.911
Late-onset sepsis, *n* (%)	10 (18.2%)	11 (17.7%)	0.951
NEC ≥ phase 2, *n* (%)	6 (10.9%)	8 (12.9%)	0.740
IVH ≥ grade 3, *n* (%)	3 (5.5%)	3 (4.8%)	0.88
ROP ≥ phase 2, *n* (%)	11 (26.8%)	18 (34.6%)	0.421

IQR, interquartile range; LISA, less invasive surfactant administration; INSURE, intubate-surfactant-extubate; MV, mechanical ventilation; PDA, patent ductus arteriosus; NEC, necrotizing enterocolitis; IVH, intraventricular hemorrhage; ROP, retinopathy of prematurity; PPV, positive pressure ventilation; dynPEEP, dynamic positive end expiratory pressure; DR delivery room.

Note: Two preterm infants died after they were diagnosed with BPD in the PPV group.

The HFO mode was used for 14 infants in the PPV group and for 16 infants in the dynPEEP group.

### 3.4. Multivariate logistic regression

We applied the demographic characteristics, dynPEEP vs. PPV and Apgar score at 1 min (variables with *P* value < 0.05) in the univariate analysis to the multivariate analysis based on logistic regression (Table 5).

**Table 5:** The multivariate analysis of the DR intubation risk based on logistic regression.

Variables	OR (95% CI)	*χ* ^2^	*P* value
dynPEEP vs. PPV	0.054 (0.012, 0.239)	14.70	0.001
Apgar score at 1 min	0.48 (0.29, 0.81)	7.49	0.0062
**Neonatal Demographics**			
Gestational age	0.34 (0.16, 0.75)	7.15	0.0075
Birthweight	1.00 (0.997, 1.005)	0.20	0.6532
Male sex	1.31 (0.41, 4.21)	0.20	0.6520
Singleton birth	1.25 (0.35, 4.53)	0.12	0.7309
Cesarean delivery	2.37 (0.61, 9.22)	1.54	0.2139
SGA	<0.001 (<0.001, >999.99)	0.0003	0.9873
DCC	1.10 (0.29, 4.19)	0.02	0.8904
**Maternal Demographics**			
Pregnancy-induced hypertension	8.29 (1.40, 49.05)	5.44	0.0197
GDM	0.38 (0.10, 1.51)	1.89	0.1693
PROM	1.24 (0.35, 4.36)	0.11	0.7418
Chorioamnionitis	0.20 (0.03, 1.30)	2.84	0.0919
Antenatal steroids (full course)	0.34 (0.07, 1.56)	1.93	0.1647
Antenatal magnesium sulfate	0.48 (0.09, 2.46)	0.78	0.3785
Antenatal antibiotics	10.42 (2.18, 49.77)	8.63	0.0033

OR, odds ratio; CI, confidence interval; SGA, Small for gestational age; DCC, delayed cord clamping; GDM, gestational diabetes mellitus; ICP, intrahepatic cholestasis of pregnancy; PROM, premature rupture of membranes; PPV, positive pressure ventilation; dynPEEP, dynamic positive end expiratory pressure; DR, delivery room.

Note: The *χ*^2^ represents the power of the association in the logistic regression model.

Logistic regression showed that dynPEEP was a protective factor compared with PPV. Moreover, the Apgar score at 1 min, GA, pregnancy-induced hypertension, and antenatal antibiotics were associated with DR intubation.

### 3.5. Subgroup analysis among infants with a ga less than 28 weeks

We further performed a subgroup analysis among infants with a GA less than 28 weeks. The maternal and neonatal demographics are shown in Supplementary Table S1. The percentage of singleton infants in the PPV group (57.7%) was significantly higher than that in the dynPEEP group (20.0%, *p* = 0.001). No significant differences in the other demographics were noted between the two groups.

Regarding the primary outcomes, the DR intubation rate was higher in the PPV group (84.6%) than in the dynPEEP group (53.3%, *p* = 0.008) (Table 2). No significant differences in the BPD rate were noted between the two groups.

With regard to the secondary outcomes (Supplementary Table S2), the DR chest compression rate was higher in the PPV group (23.1%) than in the dynPEEP group (0.0%, *p* = 0.000), and the lactate levels were higher in the PPV group (3.8 (2.0, 6.0)) than in the dynPEEP group (2.3 (1.4, 3.3), *p *= 0.013). No significant differences in the other secondary outcomes were noted.

## 4. Discussion

Clinical data regarding the effect of dynPEEP on both DR and NICU outcomes are still lacking. We conducted this retrospective cohort study to compare dynPEEP vs. PPV. We showed in this study that the dynPEEP strategy with a PEEP level of 8–15 cmH_2_O decreased the DR intubation rate. However, the dynPEEP strategy showed no impact on the BPD and/or mortality rates. In addition, the dynPEEP strategy is feasible and might represent an alternative respiratory strategy to PPV in the DR.

Endotracheal intubation is an emergency treatment for some preterm infants. Intubation is associated with increased BPD and mortality rates (27). Therefore, the dynPEEP strategy could theoretically decrease the BPD rate. In contrast, the dynPEEP strategy showed no impact on the BPD and mortality rates. We hypothesize that BPD is a multifactorial condition with antenatal genetic and environmental factors; thus, respiratory support in the DR is not the only risk factor. In addition, approximately half of the included infants had a GA > 28 weeks, among whom the BPD and mortality rates were relatively lower. This finding might also explain the lack of significant differences in the outcomes. However, when we performed a subgroup analysis among infants with a GA less than 28 weeks, we still found no differences between the two groups. The twofold higher rate of multiple births in the dynPEEP group could also partially explain the lack of difference in the BPD rate between the two groups. Moreover, as the sample size was small, the statistical analysis may not have been sufficiently statistically powered to draw a conclusion. Notably, although there was no significant difference, the rate of systemic dexamethasone decreased from 15.4% in the PPV group to 2.2% in the dynPEEP group among infants with a GA less than 28 weeks (Supplementary Table S2), which was a sevenfold decrease. We suspected that this phenomenon might explain the lessened pulmonary morbidity during the second epoch. Nevertheless, a larger, randomized control trial (RCT) is needed to evaluate the effect of the dynPEEP strategy on BPD prevalence.

Although the dynPEEP strategy could decrease the DR intubation rate, the DR intubation rate in the PPV group was 80%, which was considerably greater than those among VPIs (17%) and EPIs (36%), as described in the Background section. These data were obtained from all the VPIs and EPIs in our hospital. Nevertheless, we excluded infants supported only with PEEP levels of 6–8 cmH_2_O (*n* = 240), as shown in Figure 1. We presumed that this group of infants might have more mature lungs than those who needed dynPEEP or PPV. Therefore, the DR intubation rate in the PPV group was much higher. Consistent with the lower DR intubation rate, the chest compression rate and the percentage of 5 min Apgar scores < 5 were also lower in the dynPEEP group. Consequently, the rate of the LISA method and the percentage of infants with noninvasive respiratory support in the dynPEEP group during the transfer from the DR to the NICU were higher. Due to higher PEEP levels in the dynPEEP group, the PIP levels were lower than those in the PPV group. Given that a *ΔP* was noted in the PPV group, which could lower the CO_2_ levels, the PaCO_2_ values were higher in the dynPEEP group. Interestingly, lower lactate levels were noted in the dynPEEP group, which might be explained by the enhanced alveolar recruitment and oxygenation in the dynPEEP group. However, the PaO_2_ and *P*/F values were not significantly different between the two groups. This phenomenon should be considered in future studies.

There are concerns that higher PEEP levels could overexpand the lungs, thereby increasing the risk of pneumothorax and causing lung injury (17). The SAIL trial showed that death at less than 48 h of age occurred in 16 infants (7.4%) in the SLI group vs. 3 infants (1.4%) in the PPV group. We found no significant adverse events in the dynPEEP group in our study, but the rates of deaths at less than 48 h of age were 6.5% in the dynPEEP group and 3.6% in the PPV group. As seen from the above data, there was a higher mortality rate in the PEEP group than that in the PPV group both in our study and in the SAIL trial. Given that this was a single-center retrospective study with a small sample size, it is difficult to draw a conclusion regarding the safety of the dynPEEP strategy. A well-designed prospective study is needed to evaluate the safety of the dynPEEP strategy with SLI or PPV.

The European Guidelines on RDS management suggest that routine use of positive pressure breaths should be discouraged (9, 28). However, for babies who remain apneic or bradycardic, gentle PPV may be needed. Herein, we demonstrated that the dynPEEP strategy decreased the DR intubation rate compared to the PPV strategy. Therefore, the dynPEEP strategy might represent an alternative respiratory strategy to PPV in the DR.

A randomized controlled, multinational, multicenter trial has been ongoing since May 2021 (29). This is a two-arm study comparing the dynPEEP strategy of 8–12 cmH_2_O with the standard PEEP strategy of 5–6 cmH_2_O in the DR. The aim of the POLAR trial and our study is to compare the optimal amount or level of PEEP to give at birth and its consequential outcomes. Although the POLAR trial also focuses on the effect of dynPEEP, the PEEP range in the dynPEEP group is between 8 and 12 cmH_2_O, as mentioned above, which is different from that used in our study. One of the inclusion criteria in the POLAR trial is that infants receive respiratory intervention at birth with CPAP and/or PPV in the DR. Thus, infants in the dynPEEP group might also receive PPV, which differs from our study design. In addition, the primary outcome of the POLAR trial is the prevalence of the composite outcome of either death or BPD, as assessed by a standard oxygen reduction test, which is different from the BPD definition in our study. To date, the POLAR trial has not posted results on ClinicalTrials.gov and is currently recruiting participants. We believe that the results from the POLAR trial would be more convincing than our study because it is a prospective and multicenter RCT.

Given that this was a retrospective cohort study, some limitations should be noted. First, given the retrospective design, there may be confounding factors (twin differences, gestation, unknown, etc.) and bias, and a prospective RCT is needed to make categorical statements regarding safety or benefit. Clinicians might have been more likely to intubate the infants in the PPV group once PPV had started, which might be a potential bias. However, PPV was not allowed in the dynPEEP group because we were concerned that PPV would mask the lung protection of the dynPEEP method (13, 15, 30). Second, half of the included infants had a GA > 28 weeks; thus, the requirement for early respiratory support and the BPD and mortality rates were relatively low. This might explain the lack of significant differences in the outcomes. Although a stratified analysis among infants less than 28 weeks gestation still showed no significant difference, the small sample size might not have been sufficiently statistically powered. Furthermore, the BPD assessment had limitations due to missing data (infants were transferred to another hospital). Third, the lack of systematic assessment of compliance with dynPEEP in the DR was another limitation. Finally, the sample size was relatively small, as this was a time-based feasibility sample, and the multiple secondary statistical analyses made in this study limited the value of significant results.

## 5. Conclusions

Our study showed that the dynPEEP strategy was feasible for preterm infants with a GA < 30 weeks in the DR. Although the dynPEEP strategy with a PEEP level of 8–15 cmH_2_O could decrease the need for DR intubation, decreased BPD and/or mortality rates were not observed, and the corresponding measurable clinical outcomes were not approved. The dynPEEP strategy might be an alternative respiratory strategy in the DR instead of PPV. Because another RCT for preterm infants of less than 32 weeks GA in the DR was conducted in our hospital, we could not perform a dynPEEP RCT. A larger, multicenter RCT including more preterm infants with GAs less than 30 or 28 weeks is needed to evaluate the complete effects of the dynPEEP strategy. Above all, the dynPEEP strategy in the DR is worth further exploration.

## Data Availability

The original raw data (individual de-identified subject data) can be obtained and reviewed by email correspeondence to [YW, e-mail address: bird8227@163.com] upon reasonable request.
