# Supplementary material for: Individualized dynamic PEEP (dynPEEP) vs. positive pressure ventilation in delivery room management: A retrospective cohort study
Source: Front Pediatr. 2023 Jan 11;10:1007632. doi: 10.3389/fped.2022.1007632 (PMC9874145; doi:10.3389/fped.2022.1007632)
Supplement: Supplementary file 1 [file Table1.docx]

**Table 1.** Baseline Maternal and Neonatal Demographic and Clinical Characteristics

|  | PPV  (n = 55) | dynPEEP  (n = 62) |
| --- | --- | --- |
| **Neonatal Demographics** | | |
| Gestational age, (*x* ± SD) | 27.7 ± 1.6 | 27.2 ± 1.5 |
| Birthweight, (*x* ± SD) | 1050 ± 237 | 997 ± 239 |
| Male sex, n (%) | 34 (61.8%) | 30 (48.4%) |
| Singleton birth, n (%) | 35 (63.6%) | 14 (22.6%) * |
| Cesarean delivery, n (%) | 39 (70.9%) | 35 (56.5%) |
| SGA, n (%) | 2 (3.6%) | 1 (1.6%) |
| DCC, n (%) | 35 (63.6%) | 47 (75.8%) |
| **Maternal Demographics** | | |
| Pregnancy-induced hypertension, n (%) | 12 (21.8%) | 9 (14.5%) |
| GDM, n (%) | 14 (25.5%) | 16 (25.8%) |
| ICP, n (%) | 0 | 0 |
| PROM, n (%) | 16 (29.1%) | 19 (30.6%) |
| Chorioamnionitis, (n) (%) | 4 (7.3%) | 7 (11.3%) |
| Antenatal steroids (full course), n (%) | 29 (52.7%) | 41 (66.1%) |
| Antenatal [magnesium sulfate](D:/Dict/9.0.1.1/resultui/html/index.html#/javascript:;), n (%) | 37 (67.3%) | 45 (72.6%) |
| Antenatal antibiotics, n (%) | 18 (32.7%) | 27 (43.5%) |

* : versus PPV group, *P* <0.01.

IQR, interquartile range; SGA, Small for gestational age; DCC, delayed cord clamping; GDM, gestational diabetes mellitus; ICP, intrahepatic cholestasis of pregnancy; PROM, premature rupture of membranes; PPV, positive pressure ventilation; dynPEEP, dynamic positive end expiratory pressure.
