# Supplementary material for: Individualized dynamic PEEP (dynPEEP) vs. positive pressure ventilation in delivery room management: A retrospective cohort study
Source: Front Pediatr. 2023 Jan 11;10:1007632. doi: 10.3389/fped.2022.1007632 (PMC9874145; doi:10.3389/fped.2022.1007632)
Supplement: Supplementary file 2 [file Table2.docx]

**Table 2.** The Primary Outcomes of Preterm Infants with Gestational Age Less Than 30 Weeks Who Received PPV or DynPEEP in the DR

|  | PPV  (n = 55) | dynPEEP  (n = 62) | *P* value |
| --- | --- | --- | --- |
| **Primary Outcomes** | | | |
| DR intubation rate, n (%) | 44 (80.0%) | 28 (45.2%) | 0.000 |
| BPD, n (%) | 14/43 (32.6%) | 13/45 (28.9%) | 0.709 |
| ***Subgroup analysis (GA less than 28 weeks)*** | (n = 26) | (n = 45) |  |
| DR intubation rate, n (%) | 22 (84.6%) | 24 (53.3%) | 0.008 |
| BPD, n (%) | 10/18 (55.6%) | 13/29 (44.8%) | 0.474 |

DR, delivery room; BPD, bronchopulmonary dysplasia; PPV, positive pressure ventilation; dynPEEP, dynamic positive end expiratory pressure.

Note:

For the calculation of BPD, the denominator indicates the number of preterm infants who survived at 36 GA in our NICU. Those who died or were transferred to another hospital prior to assessment were excluded. Both the number of infants with the outcome and the number assessed are shown.
